# Splenic Hydatid Cyst Presenting As Non-cirrhotic Portal Hypertension: A Rare Case Report

**DOI:** 10.7759/cureus.60025

**Published:** 2024-05-10

**Authors:** Faizanulla Khan, Harshitha Reddy, Priyanka K Negandhi, Chaitanya Kumar Javvaji, Sunil Kumar

**Affiliations:** 1 Internal Medicine, Jawaharlal Nehru Medical College, Datta Meghe Institute of Higher Education and Research, Wardha, IND; 2 Pediatrics, Jawaharlal Nehru Medical College, Datta Meghe Institute of Higher Education and Research, Wardha, IND

**Keywords:** echinococcus, splenectomy, non cirrhotic portal hypertension, spleen, hydatid cyst

## Abstract

Hydatid cystic disease, also called cystic echinococcosis, arises from *Echinococcus*, a tapeworm infestation. It results in developing cysts primarily in the liver, although they can also occur in other organs. While the spleen is an uncommon site for cyst formation, it can still be affected. These infections are more prevalent in rural and underdeveloped regions, particularly among individuals involved in livestock rearing and animal care. The case we came across was of a 32-year-old female from a rural background engaged in animal handling and farming. She presented to our hospital with left hypochondriac pain, decreased appetite, and generalized weakness, but the patient had a history of two episodes of melena, which was self-limiting. Subsequent investigations revealed a diagnosis of splenic hydatid cyst with perisplenic collaterals and cystic compression of the splenic vein, causing symptoms of non-cirrhotic portal hypertension. Here, we present a unique case of splenic hydatid cyst leading to non-cirrhotic portal hypertension. This rare presentation poses diagnostic challenges and emphasizes the importance of considering parasitic infections in differential diagnoses.

## Introduction

The primary cestode worm responsible for causing hydatid disease (HD) is *Echinococcus granulosus* or dog tapeworm. The liver involvement is caused by hematogenous spread, and the lungs are the second most commonly involved organ. Splenic hydatidosis is a rare disease after the infestation of the metacestode form of *Echinococcus granulosus*, with a reported prevalence ranging from 0.9% to 4% [1].

Splenomegaly, characterized by an enlarged spleen, can stem from various underlying causes. These include bacterial, viral, or parasitic infections like malaria, schistosomiasis, and visceral leishmaniasis. In addition, inflammatory conditions, such as sarcoidosis and blood disorders like leukemia and lymphoma, may also contribute to splenomegaly [2]. Non-cirrhotic portal hypertension (NCPH) can also lead to splenomegaly as a consequence of increased pressure in the portal vein system [3].

Typically, these conditions remain asymptomatic for an extended duration and are often discovered incidentally during routine imaging procedures. However, in some instances, symptoms may manifest. Splenic hydatid cysts can present as abdominal discomfort, loss of appetite, and a palpable mass in the left upper quadrant, which strongly favors its possibility. While varices as a consequence of portal hypertension are listed as long-term sequelae of HD in literature, reported findings and case numbers are relatively limited compared to other complications such as bursting into the biliary system and pleural and peritoneal spaces [4]. These latter complications have been extensively sought after and are under critical consideration.

## Case presentation

We came across a case of a 32-year-old female from a rural area, a farmer by profession associated with animal handling and livestock management, including dogs and sheep, in her native village, who presented to our hospital with complaints of left-sided hypochondriac discomfort and pain for two weeks and was associated with decreased appetite for 10 days.

On general examination, the patient had signs of no pallor, icterus, cyanosis, clubbing, lower limb edema, and generalized lymph node enlargement. The patient's blood pressure was 110/70, pulse rate was 88 beats per minute, and respiratory rate of 12 per minute. She has no evidence of systemic hypertension, diabetes mellitus, bronchial asthma, or thyroid disorder. An abdominal examination reveals splenomegaly. Other systemic examinations are unremarkable.

His routine laboratory parameters have been highlighted in Table 1.

**Table 1:** Laboratory investigations of the patient

Laboratory parameters	Patient value	Reference range
Hemoglobin	11.8	12-15.0 g/dL
Total leucocyte count	8,400	4,000-10,999 mm³
Platelet count	15,000	145,000-460,000 mm³
Mean corpuscular volume	80.5	78.8-100.02 fL
Urea	23.2	9.2-20.22 mg/dL
Creatinine	0.6	0.61-1.12 mg/dL
Sodium	135.5	136-145.8 mmoL/L
Potassium	4.42	3.30-5.0 mmoL/L
Alkaline phosphatase	123.3	35.5-126.5 U/L
Alanine transaminase	27.2	<48 U/L
Aspartate transaminase	28.3	18.2-59.6 U/L
Total protein	7.40	6.48-7.78 g/dL
Albumin	3.62	3.35-4.8 g/dL
Total bilirubin	0.41	0.18-1.21 mg/dL
Globulin	4.01	2.28-3.45 g/dL
Erythrocyte sedimentation rate	82	<15 mm/hour
Activated partial thromboplastin clotting time	30.66	29.5 control
Prothrombin time	11.8	11.89 control
International normalized ratio	1.01	0.80-1.21

**Figure 1:**
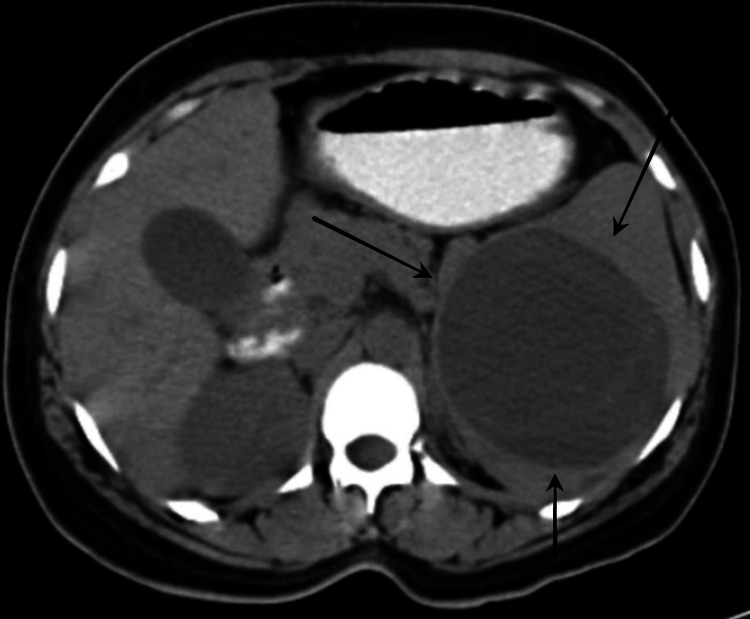
Contrast-enhanced computed tomography of the abdomen revealing a cystic lesion in the spleen (black arrows)

The patient was evaluated for abdominal pain. A contrast-enhanced computed tomography (CT) of the abdomen and pelvis was made, suggesting a well-defined cystic mass in the splenic parenchyma (Figure 1). 

**Figure 2:**
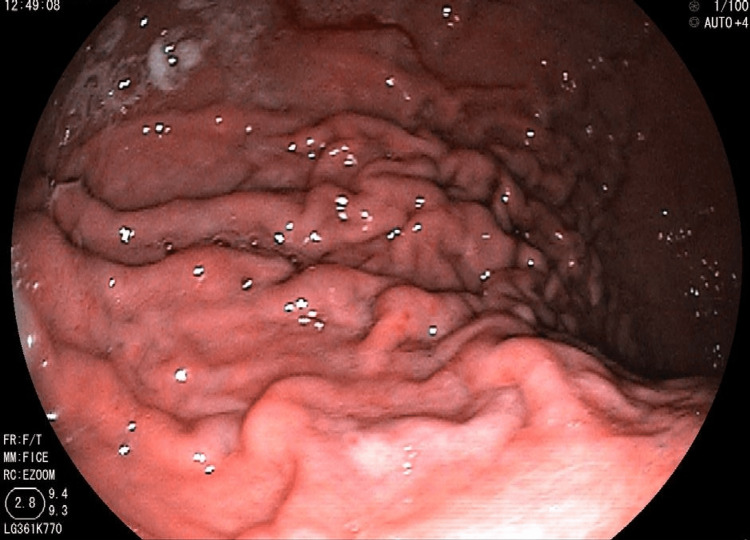
Normal upper gastrointestinal endoscopy of the patient

Upper gastrointestinal endoscopy was done and was normal (Figure 2).

The 400 mg tablet of Albendazole twice a day was initiated and continued for two weeks. Vaccinations for pneumococcus, meningococcus, and *Haemophilus influenzae* were administered two weeks before the procedure. A total abdominal splenectomy was done. The patient was symptomatically better post-surgery and was discharged on day 7 postoperatively.

## Discussion

Human infections with *Echinococcus* are attributed to four distinct species. The most commonly encountered ones, cystic echinococcosis and the HD, involving the lung stem from *Echinococcus granulosus* and *Echinococcus multilocularis*, respectively. *Echinococcus* exhibits a life cycle involving two hosts: an intermediate host, which includes animals such as sheep, goats, or swine, and a definitive host, typically dogs. Humans serve as incidental hosts in the transmission cycle and are not actively involved [5]. The tapeworm consists of segments called proglottids, which produce eggs containing embryos, which are also called oncosphere. These proglottids are usually excreted in feces by the host, i.e., a dog, into the environment, where they can infect both human accidental hosts and susceptible intermediate hosts. When the hosts eat these up, the oncosphere hatches out and penetrates the intestine's inner lining, entering the bloodstream. Subsequently, they move toward the hepatic system. Within a few days, fluid accumulates, and a cystic lesion forms, eventually developing into a metacestode or hydatid cyst in the liver. Over time, the cyst expands at a rate of 0.3-1.2 cm per year as it accumulates numerous layers over it [6]. As the echinococcosis life cycle necessitates the involvement of two animal species, direct transmission of the disease among humans is not feasible.

Cystic echinococcosis has the potential to infect any organ. However, the liver and lungs are the primary sites where the larvae typically settle. Larvae that pass through the intestinal wall via the portal circulation often become trapped in the liver, where cyst formation occurs [5]. The spleen is rarely involved in the tapeworm infestation, and the primary lesion is seen in the liver and lungs. However, splenic disease can occur when the tapeworm reaches the spleen via the arterial conduit, where the parasite's eggs bypass the liver-lung barrier. The primary foci of the spleen may travel via the artery after passing through the lung and liver.

Secondary spleen infection may also develop from the parasite's retrograde dissemination via splenic and portal veins, surpassing the lungs and liver [7]. It typically occurs following systemic dissemination or spread to the intraperitoneal structures after a hepatic hydatid cyst rupture. The development of portal hypertension in splenic hydatid cysts occurs through several mechanisms.

Mechanical obstruction of the splenic vein by the enlarging cyst can impede blood flow, leading to increased pressure within the portal venous system. Additionally, inflammatory responses triggered by the parasitic infection can contribute to portal vein thrombosis and subsequent portal hypertension. Furthermore, retrograde dissemination of parasites via splenic and portal veins can exacerbate portal hypertension, particularly following systemic dissemination or rupture of hepatic hydatid cysts [8]. NCPH encompasses a diverse range of diseases that are characterized by increased portal venous pressure gradient in the absence of liver parenchymal disease. It is not a common cause of portal hypertension globally, although its prevalence is significantly diverse [8].

Currently, the most valuable imaging modalities for diagnosing and evaluating localized splenic disorders are CT and ultrasonography, which improve the chances of diagnosis. The typical ultrasound appearance of HD manifests as an anechoic, smooth, round cyst. Signs of splenic hydatidosis on ultrasonography may include cyst wall layers, detachment of the cyst membrane from the outer layer, or cystic calcification, also known as the hydatid sand [9].

Management of splenic hydatid cysts presenting with NCPH involves a multidisciplinary approach. Surgical intervention, such as splenectomy or cystectomy, remains the cornerstone of treatment to alleviate portal hypertension and prevent further complications. Total splenic resection is the preferred treatment option in adults, with low recurrence rates observed post-surgery. Additionally, preoperative and postoperative medical prophylaxis using albendazole and praziquantel is recommended to minimize the recurrence [10].

## Conclusions

We successfully treated a rare case of symptomatic splenic hydatid cyst through surgical splenectomy, resulting in the patient's recovery without any complications. This case highlights the complexities of diagnosing and managing splenic cystic disease, especially when they remain asymptomatic for an extended period. Fortunately, the patient did not experience any adverse or anaphylactic reactions due to cyst rupture or surgical complications. Prompt diagnosis and treatment were achieved through collaborative efforts among the general medicine physician, radiologist, and gastric surgeon. This multidisciplinary approach proved instrumental in addressing this rare condition, ultimately enabling the patient to return to her native town following successful treatment.
